# Should we treat pediatric radiologically isolated syndrome? An 18-year follow-up case report

**DOI:** 10.3389/fneur.2023.1145260

**Published:** 2023-04-06

**Authors:** Elena Barbuti, Riccardo Nistri, Antonio Ianniello, Carlo Pozzilli, Serena Ruggieri

**Affiliations:** ^1^MS Center, S'Andrea Hospital, Sapienza University of Rome, Rome, Italy; ^2^Department of Human Neurosciences, Sapienza University of Rome, Rome, Italy; ^3^Neuroimmunology Unit, IRCSS Fondazione Santa Lucia, Rome, Italy

**Keywords:** RIS, pediatric radiologically isolated syndrome, treatment, children, multiple sclerosis

## Abstract

**Background:**

Radiologically isolated syndrome (RIS) describes asymptomatic individuals with incidental radiologic abnormalities suggestive of multiple sclerosis (MS). Much of RIS literature is about adult-onset cases. Treatment of RIS is controversial, especially in pediatric age, but early treatment in selected patients might improve long-term outcomes.

**Case presentation:**

We report a single RIS patient who followed up for 18 years in our MS center. At first, she was only monitored with follow-up MRIs. Then, as the lesion load increased, she was treated with a first-line disease-modifying treatment (DMT) reaching MRI stability.

**Conclusion:**

This report highlights how treatment can be an appropriate choice in pediatric forms of RIS.

## 1. Background

The term radiologically isolated syndrome (RIS) was coined by Okuda et al. (1) to describe patients with magnetic resonance imaging (MRI) features highly suggestive of multiple sclerosis (MS) without any evidence of clinical symptoms. Within 5 years of the first evidence, approximately one-third of patients with RIS develop neurological symptoms and are diagnosed with MS (2). Factors predictive of conversion are infratentorial or spinal cord lesions, gadolinium-enhancing lesions, presence of oligoclonal bands, younger age (<37), male sex, high cerebral lesion load, and abnormal visual evoked potentials (3, 4). There is a debate about whether the early initiation of DMT in RIS patients prevents conversion to clinically isolated syndrome (CIS) or MS and radiological progression and whether DMT can be considered an appropriate choice even in a patient without clinical symptoms.

## 2. Case presentation

**Figure 1:**
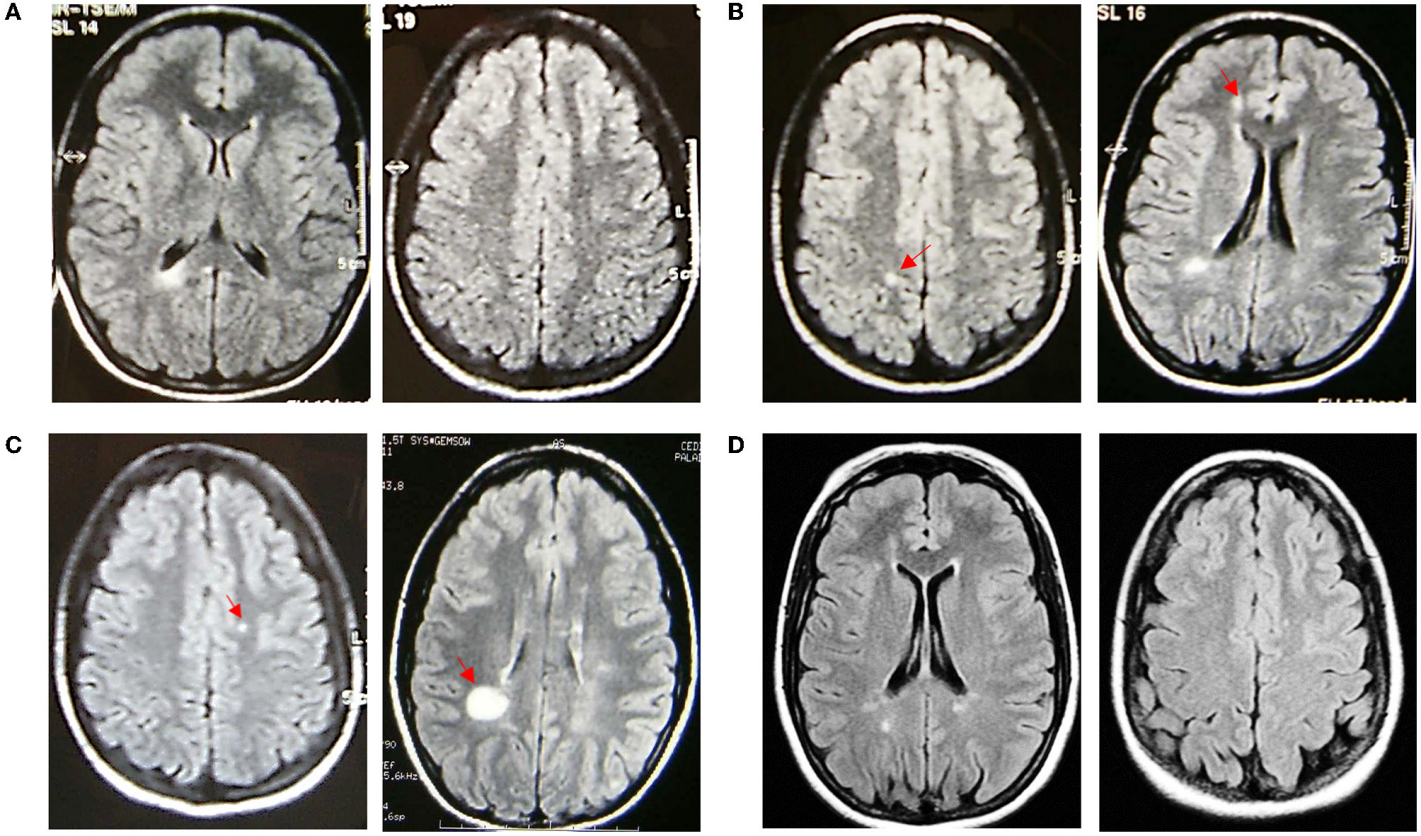
Fluid attenuated inversion recovery axial sequences of MRI scans performed in the patient over time. **(A)** Baseline MRI on 11/07/2002; **(B)** MRI on the left (10/01/2003) with a new parietal right lesion (red arrow) (new cortical frontal lesion not shown), MRI on the right (12/09/2003) with a new frontal periventricular right lesion (red arrow) (left periventricular and left temporoparietal junction new lesions not shown); **(C)** MRI on the left (29/11/2004) showing a new left corona radiata lesion (other three new lesions not shown), MRI on the right (20/05/2005) showing a new supratrigonal right lesion (red arrow); and **(D)** last MRI on 15/01/2021.

**Figure 2:**
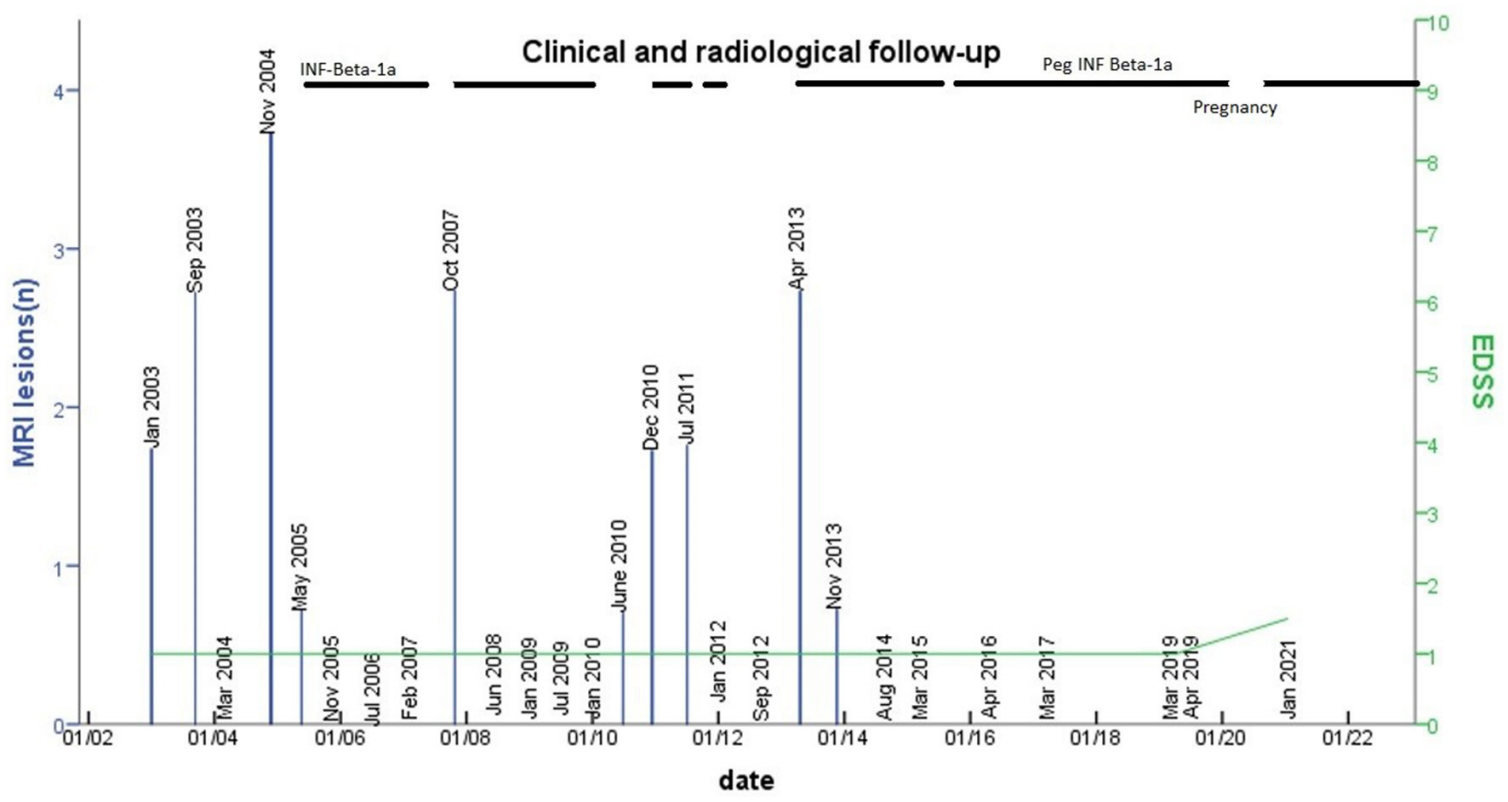
The number of new lesions in MRI follow-up and EDSS change. The number of lesions detected on MRIs performed during the follow-up is shown (blue columns). The x-axis represents time, the yaxis represents the number of new lesions on MRIs and EDSS. At the top of the table, the line stands for the time under treatment with INF beta or peginterferon. The dashed line stands for periods of irregular intake of the medication.

A 15-year-old female patient was referred to our center in July 2002 because of atypical findings on a brain MRI, requested by the general practitioner because she presented a high fever lasting several days accompanied by slight drowsiness and sleepiness. The MRI showed the altered signal intensity of the semioval center, trunk, and genu of the corpus callosum and retro-trigonal area bilaterally (Figure 1A). The neurological exam was normal, except for mild hyperreflexia on the right side [Expanded Disability Status Scale (EDSS):1.0], and we decided to monitor the evolution of the disease with a follow-up MRI. In January 2003, the brain MRI showed a new cortical parietal area and a frontal area, without contrast enhancement (Figure 1B). The cerebrospinal fluid analysis showed the presence of oligoclonal bands, with an IgG index of 2.07. The following MRIs in September 2003, November 2004, and May 2005 (Figures 1B, C) showed new small lesions (one lesion from a 2005 MRI with contrast enhancement), thus, even in the absence of any clinical onset, intramuscular INF-beta1a was prescribed on a compassionate-use basis in May 2005 to avoid further accumulation of MRI lesions. Because the drug was poorly tolerated and MRIs were stable (Figure 2), the medication was stopped in April 2007. Due to MRI activity, she restarted the treatment from November 2007 up to January 2010. During this time, annual MRIs remained stable. Then, the patient spontaneously interrupted the treatment and new lesions appeared in June 2010 and December 2010 on brain MRIs (Figure 2). Treatment was started again in December 2010, but the patient was still poorly compliant. Indeed, INF-beta1 was stopped in March 2012, and the treatment was started again in May 2013, after the detection of three new lesions on the MRI. It was switched to the pegylated form in September 2015. From then on, all brain MRIs performed until October 2019 were stable. In November 2019, she stopped the medication due to pregnancy: the baby was born without any complications. The patient was able to breastfeed for a month after the delivery; then she restarted treatment. The last brain and spine MRI performed in January 2021 was stable (Figure 1D), and the last neurological exam was normal except for mild hyperreflexia on both legs and impaired tandem walking (EDSS 1.5).

## 3. Discussion

Our patient showed several features suggesting a high risk of conversion to MS, such as oligoclonal bands, pediatric onset, high cerebral lesion load, and rapid accumulation of lesions in <3 years (5). Pediatric patients with MS have a higher annualized relapse rate and a more pronounced inflammatory pattern on the MRI compared to adults, which especially correlates with a worse prognosis and cognitive impairment (6).

Conversely, pediatric patients with MS show an enhanced capability to recover with time due to remyelination and compensatory mechanisms (7). Despite that, irreversible physical disability is reached at a younger age compared to adults (about 10 years earlier), even if in a longer time (7), and more than 30% of pediatric patients present with early cognitive deficits (impaired attention, information processing speed, episodic memory, and language), having a negative impact on school performance, daily activities over the long term (8). Indeed, pediatric multiple sclerosis is associated with reduced brain volumes compared to healthy controls at the first clinical presentation and with failure of age-expected brain growth (9). All these data indicate that early use of DMT, particularly effective in pediatric patients because of highly active inflammation, is appropriate in children to prevent disease progression, allow better recovery from relapses, and prevent brain volume reduction (10).

Even though there is no consensus on RIS, we decided to treat our patient to prevent a clinical event and long-term disability. We chose Interferon-beta (IFNB) for its good safety profile and its efficacy in pediatric patients (6, 10).

As shown in Figure 2, the appearance of new brain MRI lesions was clearly related to the period of treatment interruption, indeed MRIs were stable during treatment. Currently, two clinical trials are in progress to address the efficacy of DMT in RIS: the ARISE study: ClinicalTrials.gov Identifier: NCT02739542 (dimethyl fumarate) and the TERIS study: ClinicalTrials.gov Identifier NCT03122652 (teriflunomide). These clinical trials will have a great impact on treatment decisions in RIS, while other trials are needed in the future to test DMT in children with RIS.

## 4. Conclusion

We do believe that early treatment can be considered an appropriate choice in Pediatric RIS cases that satisfy dissemination in time and space from the McDonald revised criteria (2017), particularly considering that pediatric MS has been shown to lead to significant disability over time.

### 4.1. Patient perspective

“I feel lucky after all. At[sic] the beginning, when I was advised from[sic] my Neurologist to do these injections for these lesions I had in my head, I was not really happy. I was feeling well, so very often I forgot to take the medications. But then I began to realize that every time I stopped the injections new lesions appeared on MRI and I was concerned something bad could really happen. I trusted my doctor and took my medication like I really had multiple sclerosis. After almost 20 years I can say it was the right choice for me and for my baby.” Readapted from a talk with the patient by the authors of the Manuscript.

## Data availability statement

The original contributions presented in the study are included in the article/supplementary material, further inquiries can be directed to the corresponding author.

## Ethics statement

Written informed consent was obtained from the individual(s), and minor(s)' legal guardian/next of kin, for the publication of any potentially identifiable images or data included in this article.

## Author contributions

EB made substantial contributions to acquisition of data, investigation, and drafting the manuscript. RN and AI had a role in writing-review and editing. CP has decided to submit this manuscript for publication and supervised. SR revised the manuscript critically for important intellectual content. All authors read and approved the final manuscript.
